# Early cold stress responses in post-meiotic anthers from tolerant and sensitive rice cultivars

**DOI:** 10.1186/s12284-019-0350-6

**Published:** 2019-12-18

**Authors:** Nahuel González-Schain, Irma Roig-Villanova, Martin M. Kater

**Affiliations:** 1 Instituto de Biología Molecular y Celular de Rosario (IBR), CONICET, Facultad de Ciencias Bioquímicas y Farmacéuticas, Universidad Nacional de Rosario, Ocampo y Esmeralda, Rosario, Argentina; 2 Department of Biosciences, Università degli Studi di Milano, via Celoria 26, 20133 Milan, Italy; 3 Present address: Department of Agri-Food Engineering and Biotechnology, Barcelona School of Agricultural Engineering, UPC, Esteve Terrades 8, Building 4, 08860 Castelldefels, Spain

**Keywords:** Cold-stress, ERF, Microsporogenesis, Photosynthesis, Rice, RNA-seq, Spikelet fertility

## Abstract

**Background:**

Rice grain production is susceptible to a changing environment that imposes both biotic and abiotic stress conditions. Cold episodes are becoming more frequent in the last years and directly affect rice yield in areas with a temperate climate. Rice is particularly susceptible to cold stress during the reproductive phase, especially in anthers during post-meiotic stages which, in turn, affect pollen production. However, a number of rice cultivars with a certain degree of tolerance to cold have been described, which may represent a good breeding resource for improvement of susceptible commercial varieties. Plants experiencing cold stress activate a molecular response in order to reprogram many metabolic pathways to face these hostile conditions.

**Results:**

Here we performed RNA-seq analysis using cold-stressed post-meiotic anther samples from a cold-tolerant, Erythroceros Hokkaido (ERY), and a cold-susceptible commercial cultivar Sant’Andrea (S.AND). Both cultivars displayed an early common molecular response to cold, although the changes in expression levels are much more drastic in the tolerant one. Comparing our datasets, obtained after one-night cold stress, with other similar genome-wide studies showed very few common deregulated genes, suggesting that molecular responses in cold-stressed anthers strongly depend on conditions and the duration of the cold treatments. Cold-tolerant ERY exhibits specific molecular responses related to ethylene metabolism, which appears to be activated after cold stress. On the other hand, S.AND cold-treated plants showed a general downregulation of photosystem I and II genes, supporting a role of photosynthesis and chloroplasts in cold responses in anthers, which has remained elusive.

**Conclusions:**

Our study revealed that a number of ethylene-related transcription factors, as putative master regulators of cold responses, were upregulated in ERY providing promising candidates to confer tolerance to susceptible cultivars. Our results also suggest that the photosynthesis machinery might be a good target to improve cold tolerance in anthers. In summary, our study provides valuable candidates for further analysis and molecular breeding for cold-tolerant rice cultivars.

## Background

Rice is one of the major staple cereals in the world, providing essential caloric requirement for nearly half of the population (Khush 2005). Production of rice will need to be increased in coming decades to satisfy a steadily increasing demand from a fast-growing world population (Anderson et al. 2004). A sustainable increase in productivity requires intensified efforts to develop varieties with improved yield and greater stress tolerance. Global climate change projections pinpoint increasingly episodes of extreme temperatures. Cold temperatures affect rice growth during its whole life cycle, and especially during the seedling and reproductive stages (Andaya and Mackill 2003; Yoshida et al. 1996). In temperate regions, where rice cultivation experiences cold episodes, it is cultivated in a time-frame window of three-to-four months depending on the cultivar. In order to avoid cold episodes during seedling stages sometimes a delay in seed sowing is needed. Hence, rice plants during booting stage may encounter cold episodes at the end of the summer, highlighting the importance of planting time for the proper management of this crop (Biswas 2019).

In the last two decades an increasingly number of studies have helped to elucidate how plants respond to cold stress and the genes/proteins involved in this response. Cold is primarily sensed by cell membranes where a cold sensor triggers an influx of calcium, in order to activate downstream responses (Ma et al. 2015). The following cold signal cascade involves a number of calcium-binding proteins, mitogen-activated protein kinases and, finally, transcription factors that reprogram the transcriptome in the nucleus (Guo et al. 2018).

Cold stress produces dramatic outcomes during post-meiosis, affecting microspore generation and pollen development (Liu et al. 2019). Molecular analyses helped to understand how genomes reprogram the transcriptome and the proteins synthetized or silenced, in order to give a full molecular response to the stress. Six genome-wide transcriptional studies from cold-stressed anthers or spikelets during booting stage were reported (Bai et al. 2015; Ishiguro et al. 2014; Oda et al. 2010; Shimono et al. 2016; Suzuki et al. 2015; Yamaguchi et al. 2004). The obtained results from those studies were diverse, probably due to the use of different conditions, type and duration of treatments carried out. Nevertheless, samples analysed in those studies were collected after several days of cold stress conditions, suggesting that results obtained may not reflect the early molecular events that plants may accomplish in order to face the stress.

Here we performed RNA-seq experiments to study the genome-wide expression profiles in one-night cold-stressed post-meiotic anthers from tolerant (Erythroceros Hokkaido, ERY) and commercial susceptible (Sant’Andrea, S.AND) cultivars. Interesting genes and pathways putatively involved in cold-stress responses were identified. A detailed study of datasets obtained from this work allowed us to propose a candidate list of molecular processes and genes that might be responsible for the early differential physiological response to cold stress and, hence, be valuable for molecular breeding.

## Results

### Spikelet fertility in contrasting cultivars exposed to cold-stress

**Fig. 1:**
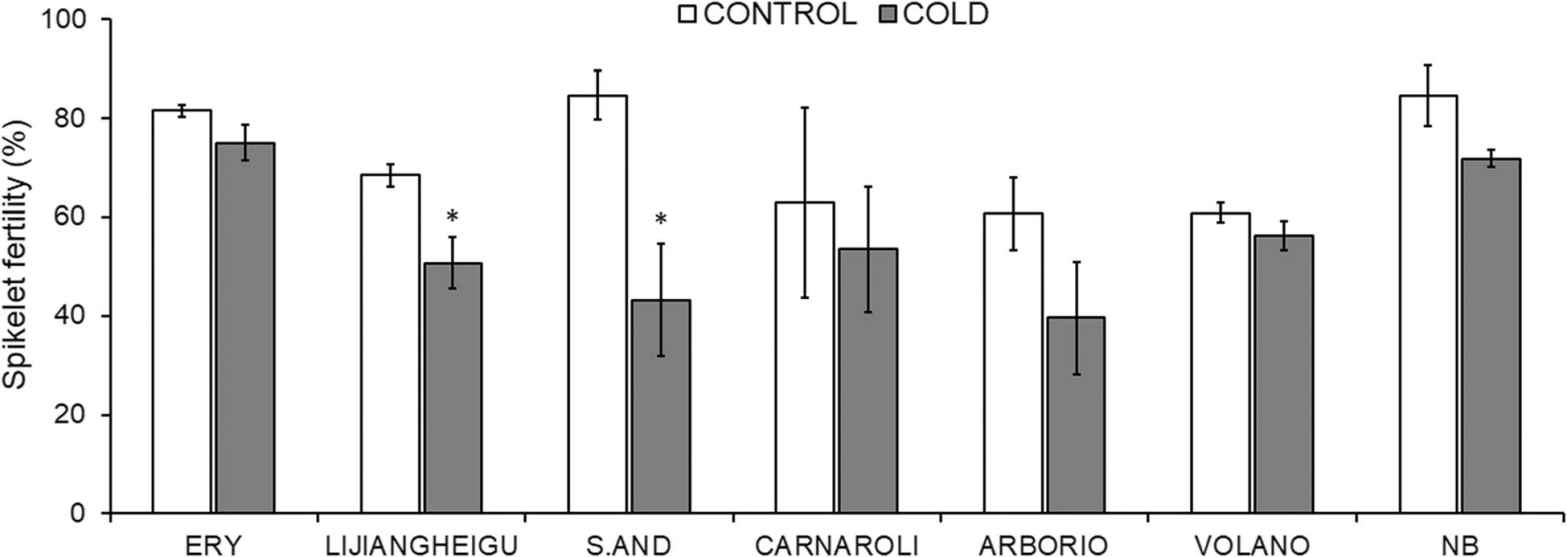
Spikelet fertility in cold-stress tolerant and sensitive cultivars. Spikelet fertility as the percentage of filled/empty spikelets after grain maturation is shown. NB, Nipponbare. Bars represent mean ± SEM of at least two biological replicas. Asterisks indicate statistically different mean values compared with the control (Student’s t-test, *p* < 0.05)

In order to study how different rice cultivars respond to low temperatures at the post-meiotic stage, the Italian accessions Arborio, Carnaroli, S.AND and Volano were chosen for analyses. Two cultivars putatively cold-tolerant, ERY and Lijiangheigu, were analysed in parallel together with the reference cultivar Nipponbare. ERY is a cultivar from Poland (Zhao et al. 2011), one of the highest latitudes in the world where rice is cultivated, while Lijiangheigu is a well-known cold-tolerant cultivar at different growth conditions (Ye et al. 2009). All cultivars tested were grown in phytotrons until pre-booting stage and subjected to cold stress (8 °C), or control conditions (22 °C) during the night in five consecutive days. Spikelet fertility was measured for all seven cultivars as the percentage of filled grains compared to total grains counted at maturity. ERY subjected to cold-stress shows spikelet fertility similar to control conditions (around 80%), behaving as a cold-tolerant cultivar in our conditions, whereas fertility in Lijiangheigu was significantly affected by cold-stress (Fig. 1). Among the Italian cultivars that we tested we found S.AND as most cold-susceptible showing a decrease of about half in spikelet fertility (Fig. 1). These results led us to choose ERY and S.AND as reference contrasting cultivars to compare physiological and molecular responses to cold-stress in detail.

### Physiological responses to cold stress in S.AND and ERY plants

As was reported previously, one of the most cold-sensitive stages during reproductive development in rice is the early microspore stage. This particular stage often correlates with the pre-booting stage but differences in reproductive development between cultivars might exist. Thus, we wanted to address the precise developmental stage that coincides with meiosis or post-meiosis to study cold responses in ERY and S.AND. We collected spikelets from both cultivars at different auricle distances (AD) between the flag leaf and the penultimate leaf (between − 5/+ 4 cm) during pre-booting stage. Transversal sections of anthers show that samples range between early post-meiotic and late vacuolated stages, corresponding to anther developmental stage 9 (Additional file 1: Figure S1a), between stage 9 and 10 (Additional file 1: Figure S1b and S1d), and stage 10 (Additional file 1: Figure S1c, S1e, and S1f) according to Zhang et al. (2011), before tapetum layer degraded. Next, we exposed ERY and S.AND panicles at AD between − 2 and + 2 cm to cold and control conditions and took samples 1, 3 and 5 days after the beginning of the treatments. Gross morphology of cold-treated panicles for both cultivars did not show any difference compared to their respective controls (Additional file 2: Figure S2A). Similar results were obtained for spikelets (Additional file 2: Figure S2b) and transverse sections of anthers (Additional file 2: Figure S2c). However, a pale green colour can be seen in cold-treated S.AND (Additional file 2: Figure S2b). In fact, many spikelets (around 10–15%) from these plants showed whitish palea and lemma (Additional file 3: Figure S3a), a phenotype that cannot be seen in control samples or in cold-treated ERY plants (Additional file 2: Figure S2). Furthermore, transversal sections of anthers from these spikelets clearly showed different levels of damaged morphology (Additional file 3: Figure S3b and S3c). These results might probably explain part of the decreased fertility observed in S.AND after cold exposure. For further experiments we only used anthers that showed normal anatomy in order to avoid biased results due to degradation of macromolecules in collected samples.

### Genes differentially expressed in post-meiotic cold-stressed anthers of S.AND and ERY cultivars

**Fig. 2:**
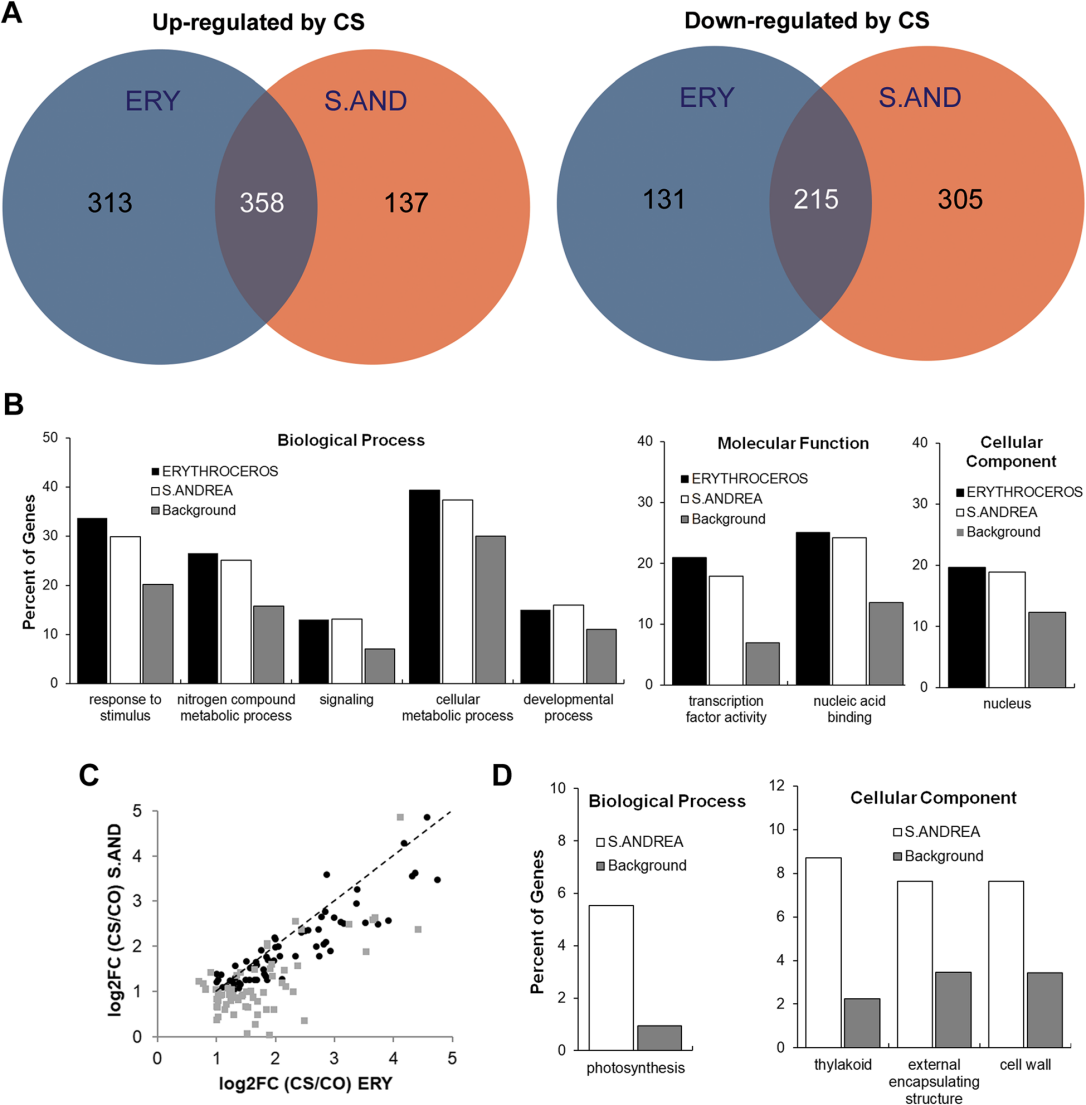
Gene ontology enrichment analysis of RNA-seq datasets. (**a**) Venn diagrams showing shared deregulated genes from cold-stress RNA-seq datasets. The most representative GO accessions are shown for ERY and S.AND upregulated (**b**) and for S.AND downregulated (**d**) genes by cold stress. Backgrounds correspond to percent of genes from the whole genome belonging to each GO accession. The full list of statistically significant (FDR < 0.05) GO accessions enriched is listed in Additional file 6: Table S2. **c**, scatterplot of log_2_FC from deregulated transcription factors in cold-stress ERY and S.AND anthers. Black circles correspond to SSTF (statistically significant by two-fold) genes in both cultivars, while grey squares correspond to genes in only one cultivar

In order to compare the early molecular responses to cold-stress from tolerant ERY and susceptible S.AND cultivars, anthers were isolated from one day cold-treated panicles specifically coinciding with post-meiotic stages as explained above (samples ERY COLD and S.AND COLD). The same type of tissues was collected from plants subjected to a normal growing temperature (24 °C) and used as controls (ERY CONTROL and S.AND CONTROL). Three biological replicates from each treatment were obtained. Total RNA was extracted from these tissues and used for Illumina RNA-seq experiments. More than 20 million reads were obtained from each of the 12 samples and mapped to the *Oryza* MSU7.0 database using the commercially available CLC Genomics Workbench (CLC bio, Aarhus, Denmark). Gene expression was quantified as reads per kilobase of coding sequence per million reads (RPKM). After normalization by total reads, statistical analyses were carried out (Baggerley’s test), and differentially expressed genes (DEGs) from control and cold samples were obtained, setting False Discovery Rate (FDR) < 0.05 and fold change (FC) > 2 as cut-offs (SSTF, statistically significant by two-fold). Nearly the same number of SSTF DEGs was obtained from each cultivar (1017 and 1015 DEGs for ERY and S.AND, respectively), although up-regulated genes were more abundant in ERY cultivar than in the cold sensitive one (Fig. 2a and Additional file 5: Table S1). Both cultivars share common cold-responsive genes showing that part of the molecular response to cold-stress is similar, as expected. However, many genes were only deregulated in one specific cultivar and might be responsible for the differential cold tolerance during reproductive stages. In order to validate RNA-seq results, six genes with altered expression levels either between controls or between control and cold-stressed samples were chosen as representatives to quantify their expression by qRT-PCR. Relative expression analysis shown in Additional file 4: Figure S4A follow the same pattern obtained by RNA-seq analysis (Additional file 4: Figure S4b), validating the genome-wide transcriptional profiling.

Next, we wanted to understand which categories were overrepresented in the DEGs lists in comparison with the whole rice genome. DEGs were further analysed for gene ontology functional annotations with agriGO analysis tools (Du et al. 2010). Up-regulated DEGs from both cultivars are enriched in genes involved in stimulus responses, nitrogen compound and cellular metabolic processes, among others, belonging to the “Biological Process” category (Additional file 6: Table S2 and Fig. 2b). Also, a significant amount of transcription factors (TFs) in those lists stand for an enrichment in the categories “Molecular Function” and “Cellular component” (Fig. 2b, Additional file 6: Table S2 and Additional file 7: Table S3). We performed a scatterplot of log_2_FC from those TFs belonging to ERY and S.AND’s DEGs lists (black circles from SSTF DEGs in both cultivars, grey squares from SSTF DEGs in only one cultivar; Fig. 2c). Strikingly, most of these transcription factor genes were more up-regulated in the cold-tolerant ERY than in the susceptible S.AND, showing a positive correlation with tolerance to cold stress. On the other hand, genes down-regulated by cold in ERY do not stand for any enriched ontological group. However, a number of thylakoid-related genes putatively involved in photosynthesis are statistically enriched in the list of cold down-regulated S.AND genes (Fig. 2d and Additional file 6: Table S2). Details of enriched gene ontology accessions and associated FDRs of DEGs are provided in Additional file 6: Table S2.

### Expression of genes involved in anther development and starch accumulation

After meiosis, there is a number of crucial biological processes taking place in anthers to ensure pollen fertility. The transport of nutrients from tapetum to microspores, degeneration of tapetal cells via programmed cell death, and pollen wall development should be well coordinated in order to produce viable pollen. Dozens of genes belonging to different pathways in these anther developmental steps have been characterized (Guo and Liu 2012; Shi et al. 2015). We reasoned that the expression of some of these genes could be deregulated between our cold tolerant and sensitive cultivars. Surprisingly, there were only three SSTF DEGs in ERY dataset, among 45 genes analyzed in both datasets (Additional file 8: Table S4 and Additional file 12: Data S1). Two of those genes showed similar FC in both cultivars while the expression of the third one, the recently characterized *TDR INTERACTING PROTEIN 3* (*TIP3*) (Yang et al. 2019), was more induced by cold stress in ERY than in S.AND cultivar (Additional file 8: Table S4 and Additional file 12: Data S1).

Starch accumulation is another crucial step in the maturation and viability of pollen grains. A cell-wall invertase and two monosaccharide transporter genes were shown to be important for starch accumulation in pollen cells and their expression were affected by cold stress (Oliver et al. 2005). We tested whether the expression of these genes, together with those reported to be involved in starch synthesis (Ohdan et al. 2005) and carbon partitioning (see references in Data S1), was affected in our datasets. We found very few SSTF DEGs in each dataset with moderated changes in their expression (Additional file 8: Table S4). The only gene consistently induced by cold stress in both cultivars was *Rice Starch Regulator 1* (*RSR1*), an AP2/EREBP transcription factor proposed to be a starch biosynthesis regulator (Fu and Xue 2010). On the other hand, only one gene, described as starch synthase IVa (Ohdan et al. 2005) was induced by cold stress in ERY but remained unaffected in S.AND (Additional file 8: Table S4).

### Common molecular responses in cold-stressed rice anthers

**Fig. 3:**
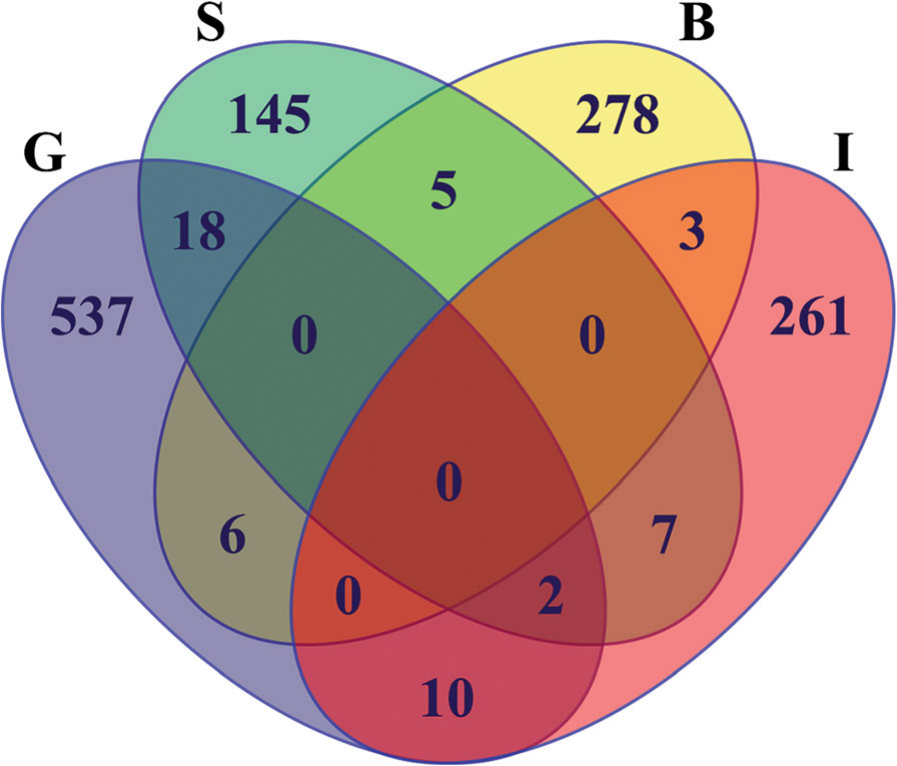
Venn diagram showing overlapped cold-stress deregulated genes in four anther datasets. Up- and downregulated genes from this work (G), (Suzuki et al. 2015) (S), (Bai et al. 2015) (B), and (Ishiguro et al. 2014) (I) were compared. Notice there are only 2 genes commonly deregulated in more than two studies

In order to elucidate common molecular responses in anthers subjected to cold-stress we compared lists of deregulated genes from our work and other published transcriptomic analysis. Six other studies have shown a variable number of differentially expressed genes in cold-stressed anthers or spikelets during young microspore stage, although cultivars, duration of treatment, temperature, etc., were very variable (Additional file 9: Table S5). From these previous studies there were only three datasets available of common cold-induced DEGs (Bai et al. 2015; Ishiguro et al. 2014; Suzuki et al. 2015) to compare with our work. Multiple comparison shows very few common genes between two datasets, where only two genes were common in three datasets: LOC_Os03g18870, encoding the heat shock protein DnaJ; and LOC_Os12g39630, encoding a calcium/calmodulin dependent protein kinase CAMK-like.49. No common deregulated genes were found in all four transcriptomic analysis (Fig. 3). These results suggest that molecular responses in cold-stress anthers are very variable and probably highly dependent on the experimental setup.

### Specific cold-induced molecular responses in ERY and S.AND

**Fig. 4:**
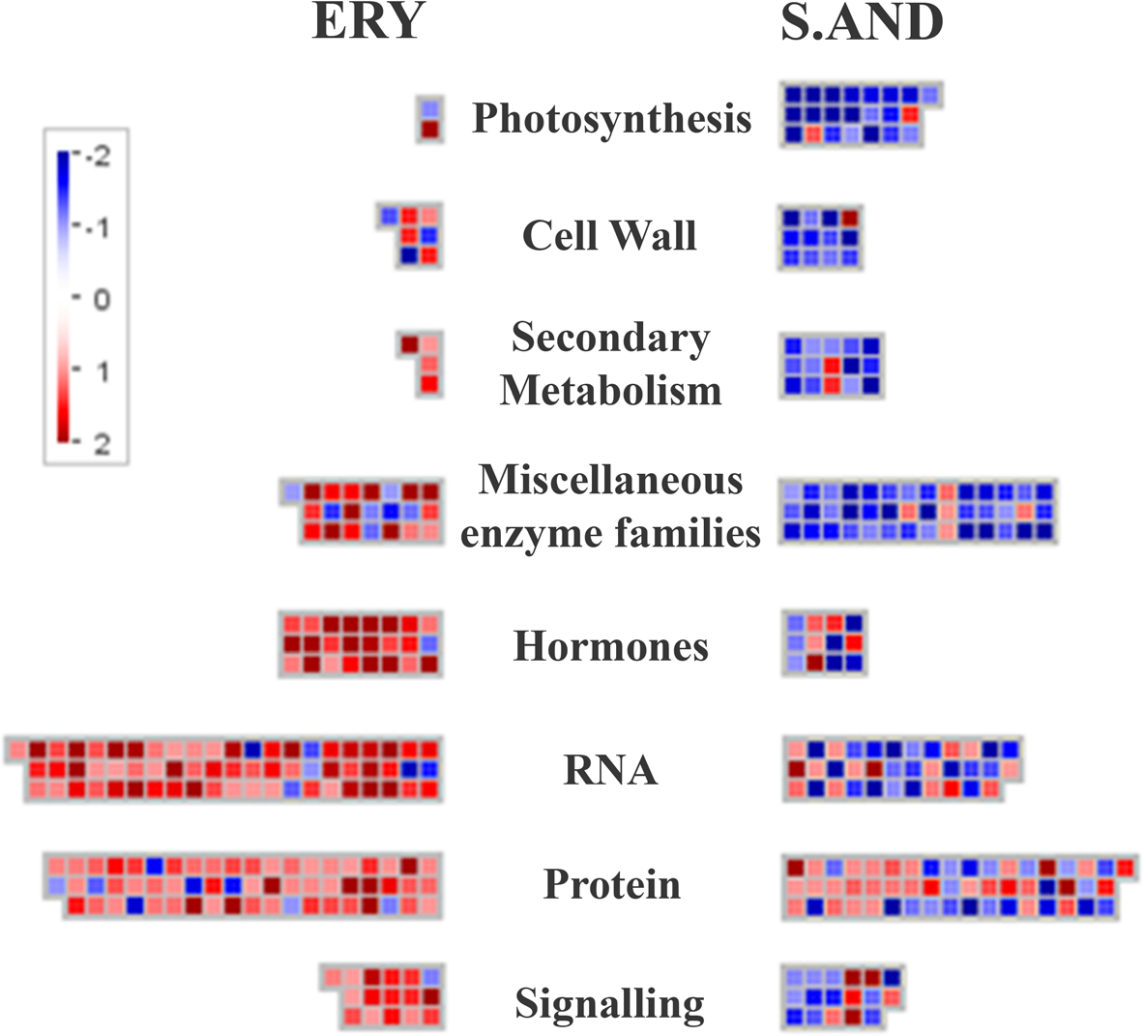
Overview of different pathways specifically deregulated in cold stress anthers. Specific up- and downregulated genes (red and blue, respectively) in cold-stress post-meiotic anthers from ERY and S.AND cultivars were analysed by Mapman software (Thimm et al. 2004)

**Fig. 5:**
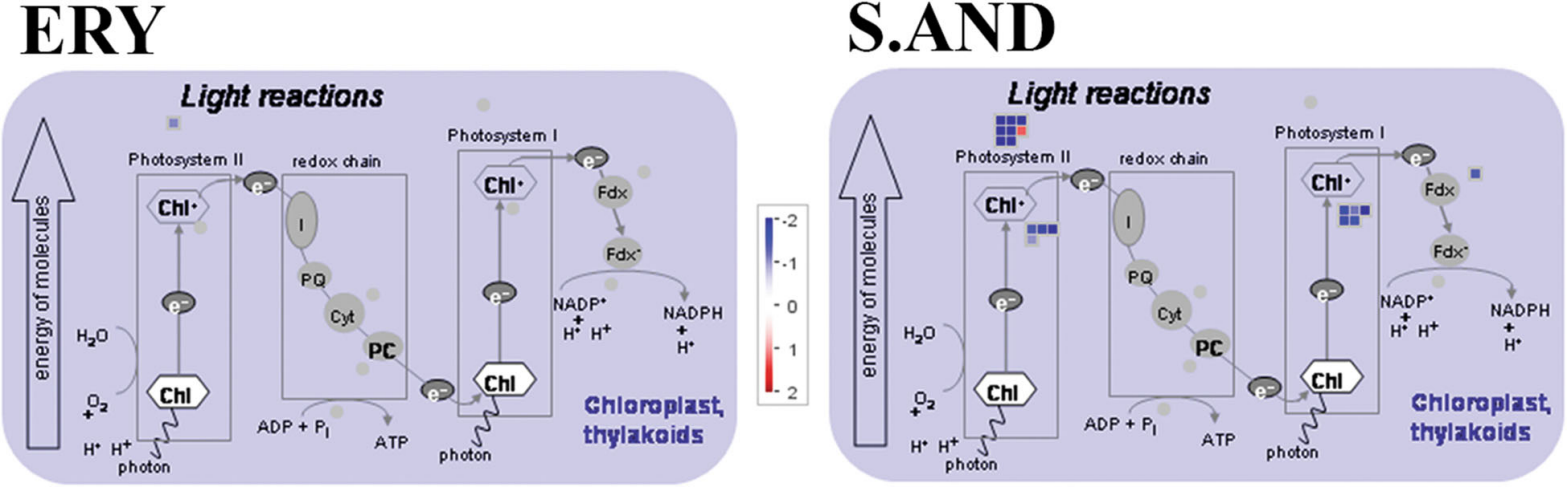
Mapman analysis of cold-stress deregulated genes involved in photosynthesis. Specific up- and down-regulated genes (red and blue, respectively) in cold-stress post-meiotic anthers from ERY and S.AND cultivars were analysed by Mapman software (Thimm et al. 2004)

To gain further insights into metabolic pathways or other biological processes differentially affected in cold-stress, ERY and S.AND samples we analysed SSTF genes specifically deregulated in each cultivar with Mapman Software (Thimm et al. 2004). Thus, a list of 444 and 442 genes specifically deregulated in ERY and S.AND, respectively, were mapped to the *Oryza* MSU7.0 database. An overview of deregulated genes falling in different hierarchical categories (Fig. 4) shows: most of the photosynthesis (bin 1), cell wall (bin 10), secondary metabolism (bin 16), and miscellaneous enzyme families (bin 26) genes were specifically downregulated in S.AND; while most of the hormones (bin 17), RNA (bin 27), protein (bin 29), and signalling (bin 30) genes were specifically upregulated in ERY. To test which bins exhibit statistically significant differences we applied Benjamini-Hochberg correction as statistical analyses, provided in Mapman software with *p* < 0.05. Two main categories were overrepresented in S.AND: bin 1.1 corresponding to “photosynthesis-light reaction”, where all genes but one is downregulated (Additional file 10: Table S6, Fig. 5) and; bin 29 corresponding to “protein”, where there is no clear trend in the regulation of these genes. Additional file 10: Table S6 also shows the two main categories overrepresented in ERY: bin 17 and 27.3.3 corresponding to “hormone metabolism” (most of them related to ethylene) and “RNA-regulation of transcription-AP2/EREBP”, respectively. Although some reports have shown, on one hand, the involvement of ethylene in male gametophyte development (Kovaleva 2011) or anther dehiscence (Rieu et al. 2003; Wang and Kumar 2007) and, on the other hand, the role of ethylene in cold stress responses, reviewed in (Kazan 2015), no reports have linked ethylene metabolism in anther tissues as part of the molecular responses to cold stress. Several genes putatively involved in ethylene biosynthesis, sensing, signal transduction and related transcription factors have been described (Rzewuski 2008). Table 1 highlights the transcriptionally active set of genes in ERY anthers during post-meiotic stages and how they respond to cold stress. Remarkably, the anther most expressed ACC oxidase genes, that catalyse a committed step in ethylene biosynthesis, OsACO2 and OsACO4 are upregulated by cold stress in ERY. Other proteins involved in the regulation of ethylene receptors (OsRTE2), and in signal transduction (OsEBF1, OsEBF2, and OsEIL2) are also upregulated. Finally, three well-known ethylene response factors are significantly upregulated as well (Sub1C, OsERF1 and osERF3). Table 1 also shows a number of ethylene-related transcription factors upregulated in ERY that might participate in the early molecular responses to cold stress.

**Table 1:** Cold-stress responses of ethylene metabolism-related genes in tolerant rice anthers. [this table should be added immediately before Discussion]

LOCUS ID	Gene	RPKM (ERY CO)	RPKM (ERY CS)	Reference
*Ethylene synthesis*				
LOC_Os03g51740	OsACS1	1.10	1.88	Rzewuski and Sauter, 2008
LOC_Os04g48850	OsACS2	0.16	0.24	Rzewuski and Sauter, 2008
LOC_Os05g10780	OsACS3	2.51	3.22	Rzewuski and Sauter, 2008
LOC_Os05g25490	OsACS4	0.01	0.02	Rzewuski and Sauter, 2008
LOC_Os01g09700	OsACS5	0.02	0.00	Rzewuski and Sauter, 2008
LOC_Os06g03990	OsACS6	20.73	25.47	Rzewuski and Sauter, 2008
LOC_Os09g27820	OsACO1	2.58	3.67	Rzewuski and Sauter, 2008
LOC_Os02g53180	OsACO2^a^	26.83	108.39	Rzewuski and Sauter, 2008
LOC_Os09g27750	OsACO3	1.18	1.09	Rzewuski and Sauter, 2008
LOC_Os11g08380	OsACO4^a^	5.86	17.86	Rzewuski and Sauter, 2008
LOC_Os05g05680	OsACO5	0.15	0.12	Rzewuski and Sauter, 2008
LOC_Os05g05670	OsACO6	0.10	0.08	Rzewuski and Sauter, 2008
LOC_Os01g39860	OsACO7	0.41	0.32	Rzewuski and Sauter, 2008
*Ethylene sensing*				
LOC_Os03g49500	OsERS1	79.28	103.50	Rzewuski and Sauter, 2008
LOC_Os05g06320	OsERS2	12.77	17.76	Rzewuski and Sauter, 2008
LOC_Os04g08740	OsETR2	27.79	40.83	Rzewuski and Sauter, 2008
LOC_Os02g57530	OsETR3	2.03	4.82	Rzewuski and Sauter, 2008
LOC_Os07g15540	OsETR4	0.28	0.54	Rzewuski and Sauter, 2008
LOC_Os01g51430	OsRTE1	14.80	11.03	Rzewuski and Sauter, 2008
LOC_Os05g46240	OsRTE2^a^	13.06	44.74	Rzewuski and Sauter, 2008
LOC_Os03g58520	OsRTE3	22.40	26.74	Rzewuski and Sauter, 2008
*Ethylene signal transduction*				
LOC_Os02g32610	OsCTR1	4.08	4.67	Rzewuski and Sauter, 2008
LOC_Os09g39320	OsCTR2	17.38	26.22	Rzewuski and Sauter, 2008
LOC_Os04g52140	OsCTR3	14.08	10.66	Rzewuski and Sauter, 2008
LOC_Os03g58060	OsEIN5	39.04	42.93	Rzewuski and Sauter, 2008
LOC_Os06g40360	OsEBF1^a^	31.00	65.78	Rzewuski and Sauter, 2008
LOC_Os02g10700	OsEBF2^a^	85.29	189.51	Rzewuski and Sauter, 2008
LOC_Os07g06130	OsEIN2	31.87	48.60	Rzewuski and Sauter, 2008
LOC_Os03g20780	OsEIL1	0.77	4.21	Rzewuski and Sauter, 2008
LOC_Os07g48630	OsEIL2^a^	78.08	220.30	Rzewuski and Sauter, 2008
LOC_Os09g31400	OsEIL3	23.82	33.25	Rzewuski and Sauter, 2008
LOC_Os08g39830	OsEIL4	6.42	6.80	Rzewuski and Sauter, 2008
LOC_Os02g36510	OsEIL5	0.32	0.07	Rzewuski and Sauter, 2008
LOC_Os04g38400	OsEIL6	3.40	3.65	Rzewuski and Sauter, 2008
*OsERFs*				
LOC_Os02g54160	OsEREBP1	377.98	404.30	Cheong et al., 2003
LOC_Os03g08460	OsEBP89	0.03	0.04	Mao et al., 2006
LOC_Os09g11460	Sub1C^a^	4.46	16.20	Fukao et al., 2006
LOC_Os12g41060	SK1/SK2	0.03	0.06	Hattori et al., 2009
LOC_Os09g28440	OsEATB	0.30	1.46	Qi et al., 2011
LOC_Os02g10760	OsWR1	194.76	180.64	Wang et al., 2012
LOC_Os08g35240	OsDERF1	0.00	0.00	Wan et al., 2011
LOC_Os01g58420	OsERF3^a^	70.93	284.72	Wan et al., 2011
LOC_Os04g52090	OsAP2–39	98.69	225.31	Wan et al., 2011
LOC_Os04g46220	OsERF1^a^	4.60	25.86	Hu et al., 2008
*Other ethylene related transcription factors (TF)*				
LOC_Os02g45010	OsbHLH076^a^	9.36	21.93	This work
LOC_Os07g28890	OsbHLH077^a^	14.40	41.89	This work
LOC_Os07g22730	OsERF136^a^	0.19	4.11	This work
LOC_Os03g08500	OsERF64^a^	265.10	1022.77	This work
LOC_Os03g08490	OsERF69^a^	0.56	6.52	This work
LOC_Os05g41780	OsERF74^a^	126.50	328.52	This work
LOC_Os09g39850	OsERF87^a^	0.38	6.54	This work
LOC_Os05g25260	OsERF56^a^	5.04	16.33	This work
LOC_Os01g49830	AP2/EREBP127^a^	6.64	19.14	This work
LOC_Os04g46440	OsERF34^a^	5.00	24.81	This work
LOC_Os04g55520	OsERF8^a^	13.93	50.32	This work
LOC_Os01g21120	OsERF68^a^	2.17	8.14	This work
LOC_Os07g42510	OsERF65^a^	2.64	9.91	This work

^a^, SSTF genes from ERY RNA-seq datasets. CO and CS, control or cold-stress treated samples, respectively

## Discussion

Cold episodes during reproductive stages in plants are becoming more frequent due to many factors in temperate regions, including climatic change, leading to important yield losses in crops. Commercial rice cultivars were selected for high yield but poor resistance to many abiotic or biotic stresses. Many molecular studies were performed in reference rice cultivars providing important insights in the response to stress, although variability lead to the notion that not always conclusions are translatable to other cultivars with agronomic relevance. This work was focused on the cold-susceptible Sant’Andrea, one of the most cultivated commercial varieties from Italy, the main producer of rice in Europe. The Erythroceros Hokkaido cultivar served as the cold-tolerant variety for comparative purposes. This cultivar grows in Poland, one of the most northern latitudes in the world in which rice is cultivated. Its highly tolerant behavior to cold stress points to a good potentiality to serve as breeding resource to introduce tolerance in cold-sensitive commercial cultivars.

Available transcriptomic studies from rice anthers subjected to cold stress led to very heterogenous results in terms of observed molecular responses, probably due to diverse conditions in which datasets were produced. In those studies, cold stress was applied at different developmental stages, at different temperatures and conditions (air or cold water). However, one of the most heterogenous factor was the duration of stress until samples were collected. This may explain the unexpectable low amount of common deregulated genes between the different studies. Many of the genome-wide transcriptomic studies were based on samples collected several days after the beginning of the cold treatment, suggesting that observed molecular changes might correspond to secondary or tertiary responses. In this study, RNA-seq data were obtained from samples subjected to cold stress during one night, where probably primary changes in the transcriptome are observed as a response to the stress applied. Changes in expression of a large number of TF encoding genes in our datasets are in agreement with that and provide a good candidate list of putative master regulators of cold responses, and may in the future be further studied to identify those genes that facilitate an increase in cold tolerance in sensitive cultivars. Remarkably, tolerant cultivar ERY showed a stronger molecular response in the activation of several TFs compared to S.AND. It might be due to a transcriptional higher rate of these master regulators, or to a faster response in the regulation of these by for instance reprogramming of the chromatin and thereby triggering the activation of the molecular machineries to successfully face stress before cell death is induced.

S.AND plants were susceptible to cold episodes during reproductive stages as it was shown for the premature degeneration of anthers and a visible decay in pigments, giving as a result a decrease in spikelet fertility. Chloroplasts might be impaired in this cultivar since results show a global decay in the synthesis of photosystem components necessary for a successful photosynthesis in anthers. This may reflect the relevance of chloroplasts for a proper male gametophyte development within anthers. The role of chloroplasts in anther development is not clear, although they are present especially in the anther epidermis, endothecium and the middle layers during microsporogenesis (Clément 2001). They may have a role in the synthesis of carbohydrates to feed tapetum, the male gametophyte and pollen grains. ERY did not show a massive downregulation of photosynthesis genes, either because chloroplasts are protected by specific metabolites and the signal triggered to degrade plastids are inhibited, or upstream master genes that regulate transcription of photosynthesis machinery are not impaired.

Finally, ethylene metabolism may be activated in ERY cold-stress anthers as a result of the transcriptional increase of several genes involved in its biosynthesis, signaling or response in the nucleus. The role of ethylene in cold-stress anthers is not known although some reports lead to the conclusion that a proper balance of its levels must be achieved for programed cell death during pollen development (Takada et al. 2006; Wei et al. 2013). The expression of a number of ethylene-related TFs is upregulated after cold treatment in ERY anthers. Only three of them (Sub1C, OsERF1 and OsERF3) are well-known TFs involved in ethylene metabolism or responsive to this phytohormone (Fukao et al. 2006; Hu et al. 2008; Wan et al. 2011). However, no evidence showing a participation of these TFs in cold stress responses has been shown yet. This work also shows more than ten putative TFs with increased expression after cold treatment in ERY, some of them with high relative expression in anthers.

## Conclusions

Our results suggest that active chloroplasts may be important during anther development to face cold episodes, making the photosynthetic machinery in anthers a target to improve cold tolerance in susceptible varieties. Our study also suggests that anther-specific ethylene metabolism, through the many transcription factors responding to cold stress in ERY, might be a good candidate for cold tolerance improvement in agronomical relevant rice cultivars as part of breeding programs or novel tools emerging for a rationale design of rice cultivars. Our studies provide an important step in the identification of possible pathways controlling cold tolerance but will of course need further validation by molecular and genetic analyses.

## Materials and methods

### Plant material and growing conditions

*Oryza sativa* subspecies with contrasting tolerance to cold stress (Erythroceros Hokkaido, Lijiangheigu, Sant’Andrea, Carnaroli, Arborio, Volano and Nipponbare) were used in the study. Plants were grown in a temperature-controlled growth chambers maintained at 26/22 °C day/night temperature and day/night relative humidity (RH) of 75–85% under LD (16/8 h light/dark) or heading-inductive SD conditions (10/14 h light/dark). Light intensity was set to ∼500 μmol/m^2^/s. Seeds were pre-germinated in seeding trays for 3–4 weeks and then seedlings were transplanted individually into pots. After 7–10 weeks, depending on the cultivar, plants were transferred to SD inductive conditions for 3 more weeks. Cold-stress treatments (8 °C) were applied only during the night period for 1, 3 or 5 days when rice plants showed particular auricle distances between flag leaf and penultimate leaf (AD) as described in results.

### Spikelet fertility

Between 2 and 13 tillers from at least three plants of each cultivar were used to estimate spikelet fertility for both control and stress treatments. At physiological maturity, the total number of spikelets and the number of grains formed were recorded and used to determine spikelet fertility. Each experiment was replicated between 2 and 4 times and mean ± SEM was obtained.

### Light microscopy

Anther developmental stages were determined by histological analysis and light microscopy. Anther samples were fixed in 3.7% formaldehyde, 5% acetic acid and 50% ethanol for 30 min with vacuum, and therefore dehydrated in ethanol series. Infiltration and embedding were carried out with Technovit acrylic resin according to the manufacturer’s instructions. 5-μm-thick sections were obtained with microtome, mounted in slides and stained with 0.1% toluidine blue. Images were captured with a Leica DM LB microscope coupled to a DFC280 camera.

### Sample collection

Between 5 and 8 plants for each biological replica were used for sample collection. For cold stress or control treatments, plants were transferred to the growth chambers when the AD matched post-meiotic developmental stage for each cultivar, corresponding to stage 10 according to Zhang et al. (2011). Spikelets from the middle part of the panicles were collected immediately after one night of corresponding treatment (when the lights of the growth chamber switched on in the morning), dissected to separate the anthers and collected in tubes suspended in liquid nitrogen. Frozen anther samples were stored at − 80 °C until further use.

### Total RNA extraction and expression analysis

Total RNA was extracted from pooled anthers by using TRIzol® (Invitrogen). Three biological replicates were collected for each treatment (Cold-stress and Control). The quality and quantity of RNA samples were assessed by gel electrophoresis and Nanodrop quantification.

cDNA synthesis and quantitative RT-qPCR were performed as described previously (Gonzalez-Schain et al. 2016). Briefly, standard RT-qPCR was carried out to test efficiency of the primers (listed in Additional file 11: Table S7) and therefore large-scale RT-qPCR was used to study the expression of deregulated genes from anther samples by using Microfluidic Dynamic Array developed by Fluidigm Corporation (Spurgeon et al., 2008). Three biological replicates with three technical replicates were performed for each sample. Data were normalized using *OsUBQ* (LOC_Os02g06640) and *NABP* (LOC_Os06g11170) genes as reference.

### Illumina sequencing

Upon treatment with TURBO DNAse I (AMBION), 4 μg of RNA from each sample were used to produce sequencing libraries with the TruSeq mRNA sample preparation kit (Illumina). Sequencing of poly(A) RNA samples was carried out in multiplex (6 samples per lane, single 50 bp reads) with Illumina Hi-seq 2000 platform at IGA Technology Services (Udine, Italy). Quality control of the raw sequence data was done using FastQC (Babraham Bioinformatics).

### Mapping of short reads, quality analysis and assessment of gene expression analysis for RNA-seq

Evaluation and processing of raw data was performed on the commercially available CLC Genomics Workbench v.4.7.1 as described previously (Gonzalez-Schain et al. 2016). Gene expression values were based on reads per kilobase of exon model per million mapped read (RPKM) values. Fold change (FC) and log_2_FC was calculated in terms of RPKM of the corresponding transcripts. To obtain statistical confirmation of the differences in gene expression, P and FDR values were computed using Baggerley’s test on expression proportions. We applied a threshold value of *P <* 0.05 and FDR < 0.05 to ensure that differential gene expression was maintained at a significant level (5%) for the individual statistical tests. Absolute FC ≥ 2 was set as threshold limit to obtain the differentially expressed genes. Raw and processed data have been deposited in NCBI’s Gene Expression Omnibus (Edgar et al. 2002) and are accessible through GEO Series accession number GSE137002 (https://www.ncbi.nlm.nih.gov/geo/query/acc.cgi?acc=GSE137002).

To gain insight into the biological processes associated with the regulated genes, we determined which GO annotation terms were over-represented, in both up- and down-regulated gene lists. Gene set enrichment analysis was performed with the agriGO database (Du et al. 2010) using the Singular Enrichment Analysis (SEA). Mapman Software (Thimm et al., 2004) was also used to get an overview of deregulated genes falling in different hierarchical categories (or bins), applying Benjamini-Hochberg correction as statistical analyses provided in the software with *p* < 0.05.

## Supplementary information

**Additional file 1: Figure S1.** Anther developmental stages in tolerant and sensitive cultivars. Transverse sections of anthers from ERY (A, C and E) and S.AND plants (B, D and F), collected at different auricle distance between flag and penultimate leaves (AD), were stained with 0.1% toluidine blue. AD = − 3, − 5, 0, − 3, 4, and 1.5 cm in A, B, C, D, E, and F, respectively.

**Additional file 2: Figure S2.** Physiological response to cold stress in tolerant and sensitive cultivars. Cold-tolerant ERY and cold-sensitive S.AND were subjected to cold stress or control conditions during 1, 3, and 5 days during post-meiotic stage. A and B, Panicles and spikelets from ERY and S.AND plants after cold or control treatments. C, transverse sections of anthers from ERY and S.AND plants after 1-day cold or control treatments (AD between − 2 and + 2 cm, corresponding to anther developmental stage 10 according to Zhang et al. (2011)) stained with 0.1% toluidine blue.

**Additional file 3: Figure S3.** Anther development is impaired in S.AND cultivar subjected to cold stress. S.AND plants were treated with cold stress for five days. A, spikelets from S.AND plants subjected to 1, 3, and 5 days of cold-stress. Notice the pale colour of palea and lemma compared to Fig. S2B. B and C, transverse sections of 1-day cold-stress anthers at stage 10, according to Zhang et al. (2011), stained with 0.1% toluidine blue, showing compromised anatomy.

**Additional file 4: Figure S4.** Validation of RNA-seq data by qRT-PCR. A, Expression analysis of 6 selected genes from RNA-seq datasets were performed by reverse transcription and real-time PCR. Data were normalized using *OsUBQ* (LOC_Os02g06640) and *NABP* (LOC_Os06g11170) as housekeeping genes and setting ERY or S.AND control samples as 1. Three biological replicates with three technical replicates were performed for each sample. Bars represent mean ± SEM. Asterisks indicate statistically different mean values compared with the corresponding control (Student’s t-test, *p* < 0.05). B, RPKMs of selected genes from RNA-seq data of ERY and S.AND samples.

**Additional file 5: Table S1.** List of DEGs from RNA-seq data.

**Additional file 6: Table S2.** Gene ontology of DEGs obtained from RNA-seq data

**Additional file 7: Table S3.** List of transcription factors upregulated in cold stress.

**Additional file 8: Table S4.** Expression values of known anther development and starch accumulation genes.

**Additional file 9: Table S5.** Transcriptomic studies from cold-stressed rice anthers.

**Additional file 10: Table S6.** List of Mapman bins, and DEGs within, specifically de-regulated in each cultivar.

**Additional file 11: Table S7.** Oligonucleotides used in this work.

**Additional file 12: Data S1.** Supplemental references for Table S4

## Data Availability

All data supporting the conclusions of this article are provided within the article and its supplementary files.

## Abbreviations

AD: Auricle distance between the flag leaf and the penultimate leaf
ERF: Ethylene Response Factor
ERY: ERYTHROCEROS HOKKAIDO
S.AND: SANT’ANDREA
TF: Transcription factor
