# Supplementary figures and images for: Early cold stress responses in post-meiotic anthers from tolerant and sensitive rice cultivars
Source: Rice (N Y). 2019 Dec 18;12:94. doi: 10.1186/s12284-019-0350-6 (PMC6920279; doi:10.1186/s12284-019-0350-6)

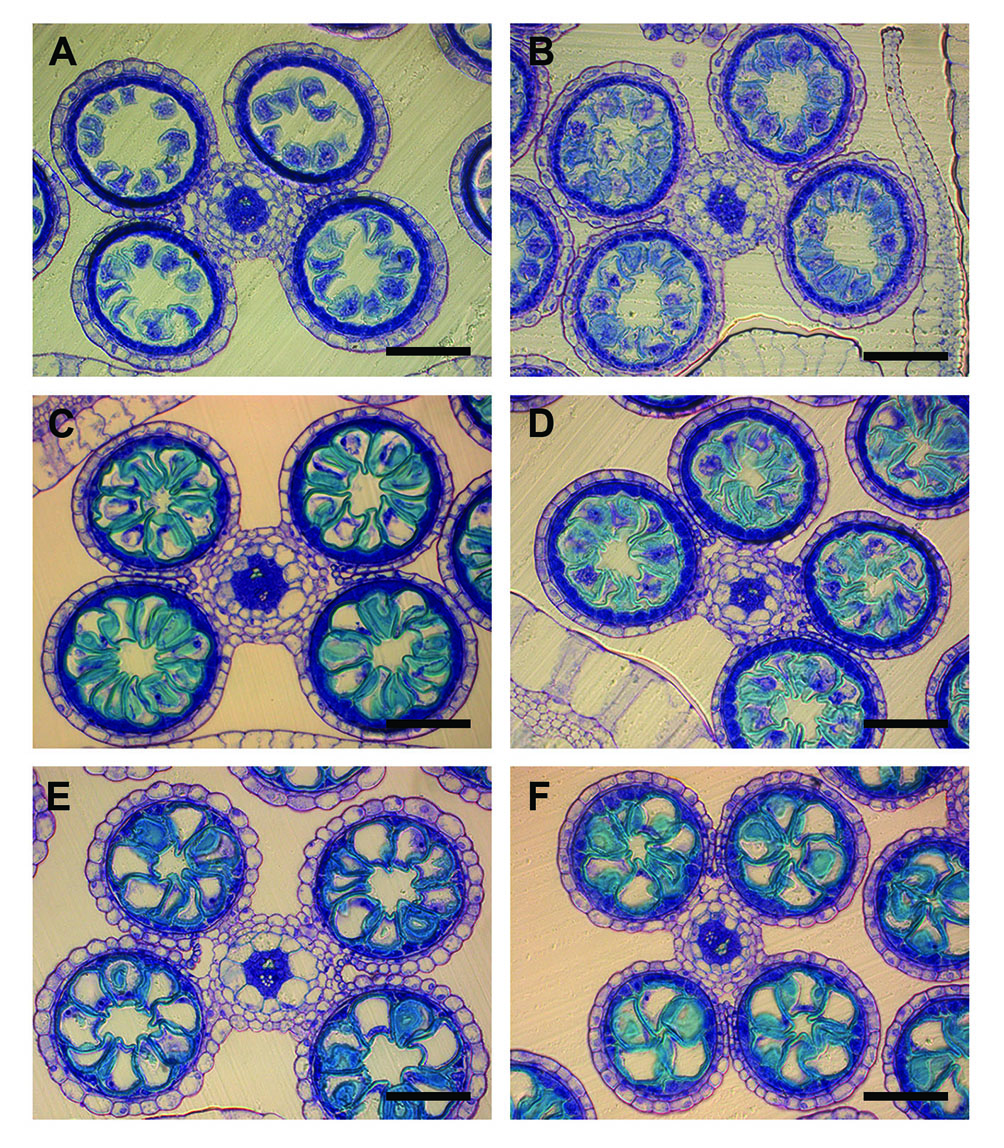

Supplement: Supplementary file 1 — Additional file 1: Figure S1. Anther developmental stages in tolerant and sensitive cultivars. Transverse sections of anthers from ERY (A, C and E) and S.AND plants (B, D and F), collected at different auricle distance between flag and penultimate leaves (AD), were stained with 0.1% toluidine blue. AD = − 3, − 5, 0, − 3, 4, and 1.5 cm in A, B, C, D, E, and F, respectively. [file 12284_2019_350_MOESM1_ESM.jpg]

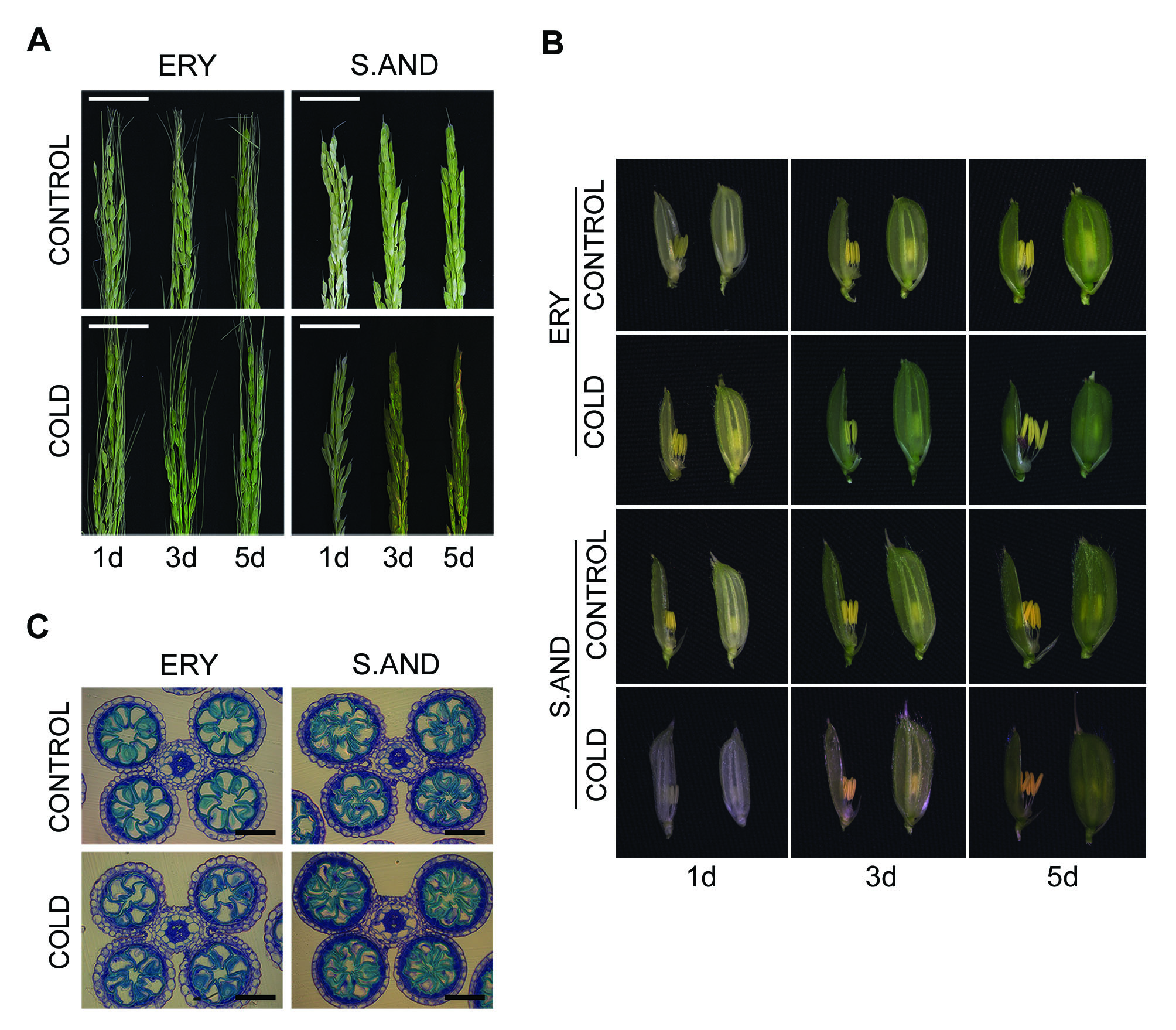

Supplement: Supplementary file 2 — Additional file 2: Figure S2. Physiological response to cold stress in tolerant and sensitive cultivars. Cold-tolerant ERY and cold-sensitive S.AND were subjected to cold stress or control conditions during 1, 3, and 5 days during post-meiotic stage. A and B, Panicles and spikelets from ERY and S.AND plants after cold or control treatments. C, transverse sections of anthers from ERY and S.AND plants after 1-day cold or control treatments (AD between − 2 and + 2 cm, corresponding to anther developmental stage 10 according to Zhang et al. (2011)) stained with 0.1% toluidine blue. [file 12284_2019_350_MOESM2_ESM.jpg]

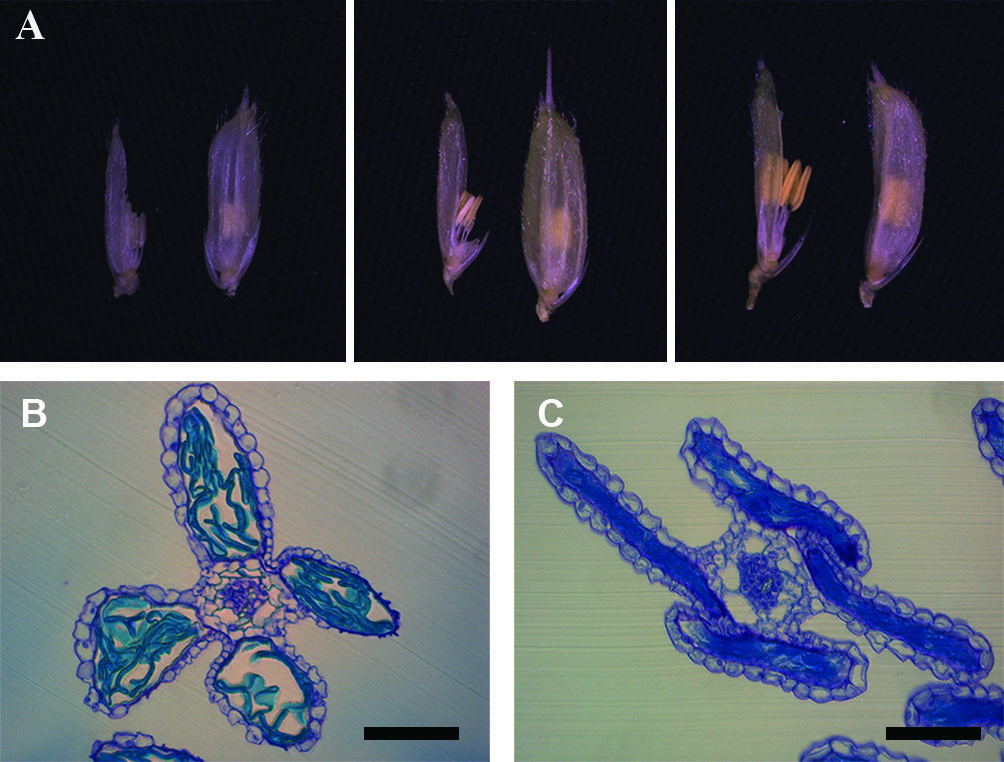

Supplement: Supplementary file 3 — Additional file 3: Figure S3. Anther development is impaired in S.AND cultivar subjected to cold stress. S.AND plants were treated with cold stress for five days. A, spikelets from S.AND plants subjected to 1, 3, and 5 days of cold-stress. Notice the pale colour of palea and lemma compared to Fig. S2B. B and C, transverse sections of 1-day cold-stress anthers at stage 10, according to Zhang et al. (2011), stained with 0.1% toluidine blue, showing compromised anatomy. [file 12284_2019_350_MOESM3_ESM.jpg]

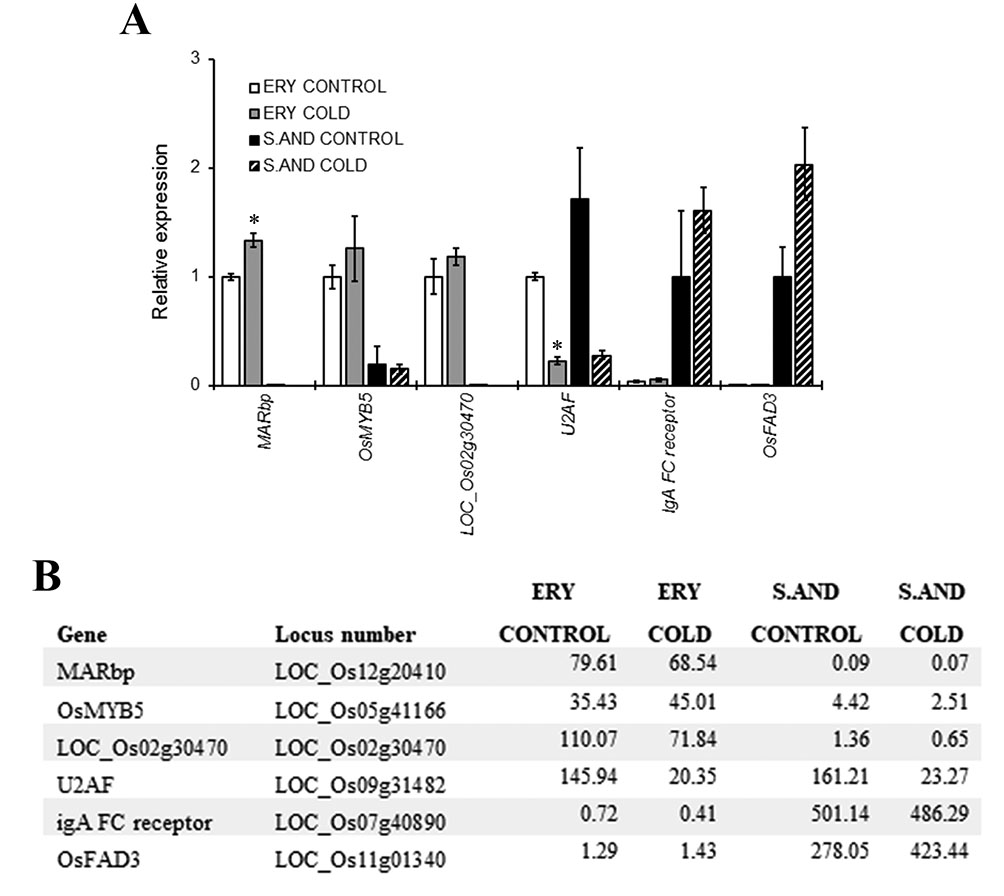

Supplement: Supplementary file 4 — Additional file 4: Figure S4. Validation of RNA-seq data by qRT-PCR. A, Expression analysis of 6 selected genes from RNA-seq datasets were performed by reverse transcription and real-time PCR. Data were normalized using OsUBQ (LOC_Os02g06640) and NABP (LOC_Os06g11170) as housekeeping genes and setting ERY or S.AND control samples as 1. Three biological replicates with three technical replicates were performed for each sample. Bars represent mean ± SEM. Asterisks indicate statistically different mean values compared with the corresponding control (Student’s t-test, p < 0.05). B, RPKMs of selected genes from RNA-seq data of ERY and S.AND samples. [file 12284_2019_350_MOESM4_ESM.tif]
