# Supplementary material for: Early cold stress responses in post-meiotic anthers from tolerant and sensitive rice cultivars
Source: Rice (N Y). 2019 Dec 18;12:94. doi: 10.1186/s12284-019-0350-6 (PMC6920279; doi:10.1186/s12284-019-0350-6)
Supplement: Supplementary file 9 — Additional file 9: Table S5. Transcriptomic studies from cold-stressed rice anthers. [file 12284_2019_350_MOESM9_ESM.docx]

| Method | Tissues collected | Treatment | Cultivars (spikelet fertility) | Reference |
| --- | --- | --- | --- | --- |
| microarray | Anthers in early- middle microspore stage | Control: 26°-20°C for 5d  Cold: 12°C for 5d  (from tetrad stage to middle microspore stage) – from AD=-7 to -10cm | Hayayuki (fertility not showed) | Yamaguchi *et al.*, 2004 |
| microarray | Anthers in uninucleate microspore stage and trinucleate pollen stage | Control: normal T (field temperature) until sampling  Cold: 19°C (cold-water system) until sampling  Sampling = ~2 months after primordial stage | Hitomebore (cold-tolerant, ~90% fertility)  Sasanishiki (cold-sensitive, ~40% fertility) | Oda *et al.*, 2010 |
| microarray | Anthers before treatment (control, C0), 1d cold (C1), 3d cold (C3) and 4d cold + 1d normal temperature (C5) | Control: 25°-19°C 0d (before treatment)  Cold: 12°C for 4d  from AD = -1 to +1cm | Nipponbare (cold-sensitive, ~30% fertility)  T65 (cold-tolerant, ~70% fertility)  Hoshinoyume (cold-tolerant, ~60% fertility)  A58 (cold-tolerant, ~70% fertility)  Silewah (cold-tolerant, ~80% fertility) | Ishiguro *et al.*, 2014 |
| RNAseq | Anthers in early microspore stage | Control: 22°C for 10d  Cold: 17.5°C for 10d  (from stage 5 to 8-9) | Y58S (cold-sensitive, ~50% fertility)  P64S (higly cold-sensitive, ~25% fertility) | Bai *et al.*, 2015 |
| microarray | Spikelets at young microspore stage | Control: 25°-20°C 5d  Cold: 12°C for 5d | Hayayuki (fertility ~80%) | Suzuki *et al.*, 2015 |
| microarray | Spikelets at young microspore stage (palea length 3-4mm) | Control: 22°C until sampling  Cold: 19°C until sampling  Sampling = ~2 months after sowing | Tohoku-PL3 (cold-tolerant, ~90%)  Akihikari (cold-sensitive, ~10%) | Shimono *et al.*, 2016 |

**Table S4. Transcriptomic studies from cold-stressed rice anthers**
