# Supplementary material for: Early cold stress responses in post-meiotic anthers from tolerant and sensitive rice cultivars
Source: Rice (N Y). 2019 Dec 18;12:94. doi: 10.1186/s12284-019-0350-6 (PMC6920279; doi:10.1186/s12284-019-0350-6)
Supplement: Supplementary file 11 — Additional file 11: Table S7. Oligonucleotides used in this work. [file 12284_2019_350_MOESM11_ESM.docx]

| LOCUS ID | Gene | Primers |
| --- | --- | --- |
| LOC_Os12g20410 | *MARbp* | Fw: 5’-TGAATGCCTACAGCGAGATG-3’  Rev: 5’-TTGGCCTCCTTTACCAAGTC-3’ |
| LOC_Os05g41166 | *OsMYB5* | Fw: 5’-TGAGCAATAGCGATGACGAG-3’  Rev: 5’-AGGCCAGTGTTCTTCTGCAC-3’ |
| LOC_Os02g30470 | *expressed protein* | Fw: 5’-GTCCTTCAGACAGCGAGAGC-3’  Rev: 5’-ATCGCTTCTCCTCAATCTGC-3’ |
| LOC_Os09g31482 | *U2AF* | Fw: 5’-AAGTACGGCGAGATCGAGAG-3’  Rev: 5’-TGATCCTCCTCCCTGAATTG-3’ |
| LOC_Os07g40890 | *igA FC receptor* | Fw: 5’-GTTTCCGAAGGTGCCTGAAC-3’  Rev: 5’-GAAGTGGAACTCAGGCTTGG-3’ |
| LOC_Os11g01340 | *OsFAD3* | Fw: 5’-AGCTTGCTTTCCTTCCTTCC-3’  Rev: 5’-AATTTGAGGGGAGGTGTTTG-3’ |
| LOC_Os02g06640 | *OsUBQ* | Fw: 5’-GAGCCTCTGTTCGTCAAGTA-3’  Rev: 5’-ACTCGATGGTCCATTAAACC-3’ |
| LOC_Os06g11170 | *NABP* | Fw: 5’-GGAATGTGGACGGTGACACT-3’  Rev: 5’-TCAAAATAGAGTCCAGTAGATTTGTCA-3’ |

**Table S6. Oligonucleotides used in this work**
