# Supplementary material for: Early cold stress responses in post-meiotic anthers from tolerant and sensitive rice cultivars
Source: Rice (N Y). 2019 Dec 18;12:94. doi: 10.1186/s12284-019-0350-6 (PMC6920279; doi:10.1186/s12284-019-0350-6)
Supplement: Supplementary file 12 — Additional file 12: Data S1. Supplemental references for Table S4 [file 12284_2019_350_MOESM12_ESM.docx]

**Supplemental References for Table S4**

Aoki, N., Hirose, T., Scofield, G. N., Whitfeld, P. R., & Furbank, R. T. (2003). The sucrose transporter gene family in rice. *Plant and Cell Physiology*, *44*(3), 223-232.

Cai, C. F., Zhu, J., Lou, Y., Guo, Z. L., Xiong, S. X., Wang, K., & Yang, Z. N. (2015). The functional analysis of OsTDF1 reveals a conserved genetic pathway for tapetal development between rice and Arabidopsis. *Science Bulletin*, *60*(12), 1073-1082.

Chen, R., Zhao, X., Shao, Z., Wei, Z., Wang, Y., Zhu, L., ... & He, G. (2007). Rice UDP-glucose pyrophosphorylase1 is essential for pollen callose deposition and its cosuppression results in a new type of thermosensitive genic male sterility. *The Plant Cell*, *19*(3), 847-861.

Fu, F. F., & Xue, H. W. (2010). Coexpression analysis identifies Rice Starch Regulator1, a rice AP2/EREBP family transcription factor, as a novel rice starch biosynthesis regulator. *Plant Physiology*, *154*(2), 927-938.

Fu, Z., Yu, J., Cheng, X., Zong, X., Xu, J., Chen, M., ... & Liang, W. (2014). The rice basic helix-loop-helix transcription factor TDR INTERACTING PROTEIN2 is a central switch in early anther development. *The Plant Cell*, *26*(4), 1512-1524.

Han, M. J., Jung, K. H., Yi, G., Lee, D. Y., & An, G. (2006). Rice Immature Pollen 1 (RIP1) is a regulator of late pollen development. *Plant and cell physiology*, *47*(11), 1457-1472.

Hu, L., Liang, W., Yin, C., Cui, X., Zong, J., Wang, X., ... & Zhang, D. (2011). Rice MADS3 regulates ROS homeostasis during late anther development. *The Plant Cell*, *23*(2), 515-533.

Ji, C., Li, H., Chen, L., Xie, M., Wang, F., Chen, Y., & Liu, Y. G. (2013). A novel rice bHLH transcription factor, DTD, acts coordinately with TDR in controlling tapetum function and pollen development. *Molecular plant*, *6*(5), 1715-1718.

Jung, K. H., Han, M. J., Lee, Y. S., Kim, Y. W., Hwang, I., Kim, M. J., ... & An, G. (2005). Rice Undeveloped Tapetum1 is a major regulator of early tapetum development. *The Plant Cell*, *17*(10), 2705-2722.

Jung, K. H., Han, M. J., Lee, D. Y., Lee, Y. S., Schreiber, L., Franke, R., ... & Hwang, I. (2006). Wax-deficient anther1 is involved in cuticle and wax production in rice anther walls and is required for pollen development. *The Plant Cell*, *18*(11), 3015-3032.

Kaneko, M., Inukai, Y., Ueguchi-Tanaka, M., Itoh, H., Izawa, T., Kobayashi, Y., ... & Matsuoka, M. (2004). Loss-of-function mutations of the rice GAMYB gene impair α-amylase expression in aleurone and flower development. *The Plant Cell*, *16*(1), 33-44.

Ko, S. S., Li, M. J., Ku, M. S. B., Ho, Y. C., Lin, Y. J., Chuang, M. H., ... & Chan, M. T. (2014). The bHLH142 transcription factor coordinates with TDR1 to modulate the expression of EAT1 and regulate pollen development in rice. *The Plant Cell*, *26*(6), 2486-2504.

Lee, S., Jung, K. H., An, G., & Chung, Y. Y. (2004). Isolation and characterization of a rice cysteine protease gene, OsCP1, using T-DNA gene-trap system. *Plant molecular biology*, *54*(5), 755-765.

Li, N., Zhang, D. S., Liu, H. S., Yin, C. S., Li, X. X., Liang, W. Q., ... & Wen, T. Q. (2006). The rice tapetum degeneration retardation gene is required for tapetum degradation and anther development. *The Plant Cell*, *18*(11), 2999-3014.

Li, H., Pinot, F., Sauveplane, V., Werck-Reichhart, D., Diehl, P., Schreiber, L., ... & Liang, W. (2010). Cytochrome P450 family member CYP704B2 catalyzes the ω-hydroxylation of fatty acids and is required for anther cutin biosynthesis and pollen exine formation in rice. *The Plant Cell*, *22*(1), 173-190.

Li, X., Gao, X., Wei, Y., Deng, L., Ouyang, Y., Chen, G., ... & Wu, C. (2011a). Rice APOPTOSIS INHIBITOR5 coupled with two DEAD-box adenosine 5′-triphosphate-dependent RNA helicases regulates tapetum degeneration. *The Plant Cell*, *23*(4), 1416-1434.

Li, H., Yuan, Z., Vizcay-Barrena, G., Yang, C., Liang, W., Zong, J., ... & Zhang, D. (2011b). PERSISTENT TAPETAL CELL1 encodes a PHD-finger protein that is required for tapetal cell death and pollen development in rice. *Plant physiology*, *156*(2), 615-630.

Li, L., Li, Y., Song, S., Deng, H., Li, N., Fu, X., ... & Yuan, L. (2015). An anther development F-box (ADF) protein regulated by tapetum degeneration retardation (TDR) controls rice anther development. *Planta*, *241*(1), 157-166.

Li, Y., Li, D., Guo, Z., Shi, Q., Xiong, S., Zhang, C., ... & Yang, Z. (2016). OsACOS12, an orthologue of Arabidopsis acyl-CoA synthetase5, plays an important role in pollen exine formation and anther development in rice. *BMC plant biology*, *16*(1), 256.

Liu, Z., Lin, S., Shi, J., Yu, J., Zhu, L., Yang, X., ... & Liang, W. (2017). Rice No Pollen 1 (NP 1) is required for anther cuticle formation and pollen exine patterning. *The Plant Journal*, *91*(2), 263-277.

Luo, H., Lee, J. Y., Hu, Q., Nelson-Vasilchik, K., Eitas, T. K., Lickwar, C., ... & Hodges, T. K. (2006). RTS, a rice anther-specific gene is required for male fertility and its promoter sequence directs tissue-specific gene expression in different plant species. *Plant molecular biology*, *62*(3), 397-408.

Moon, S., Kim, S. R., Zhao, G., Yi, J., Yoo, Y., Jin, P., ... & An, G. (2013). Rice glycosyltransferase1 encodes a glycosyltransferase essential for pollen wall formation. *Plant physiology*, *161*(2), 663-675.

Moritoh, S., Miki, D., Akiyama, M., Kawahara, M., Izawa, T., Maki, H., & Shimamoto, K. (2005). RNAi-mediated silencing of OsGEN-L (OsGEN-like), a new member of the RAD2/XPG nuclease family, causes male sterility by defect of microspore development in rice. *Plant and cell physiology*, *46*(5), 699-715.

Mu, H., Ke, J., Liu, W., Zhuang, C., & Yip, W. (2009). UDP-glucose pyrophosphorylase2 (OsUgp2), a pollen-preferential gene in rice, plays a critical role in starch accumulation during pollen maturation. *Chinese science bulletin*, *54*(2), 234.

Niu, N., Liang, W., Yang, X., Jin, W., Wilson, Z. A., Hu, J., & Zhang, D. (2013). EAT1 promotes tapetal cell death by regulating aspartic proteases during male reproductive development in rice. *Nature communications*, *4*, 1445.

Nonomura, K. I., Miyoshi, K., Eiguchi, M., Suzuki, T., Miyao, A., Hirochika, H., & Kurata, N. (2003). The MSP1 gene is necessary to restrict the number of cells entering into male and female sporogenesis and to initiate anther wall formation in rice. *The Plant Cell*, *15*(8), 1728-1739.

Ohdan, T., Francisco Jr, P. B., Sawada, T., Hirose, T., Terao, T., Satoh, H., & Nakamura, Y. (2005). Expression profiling of genes involved in starch synthesis in sink and source organs of rice. *Journal of experimental botany*, *56*(422), 3229-3244.

Oliver, S. N., Van Dongen, J. T., Alfred, S. C., Mamun, E. A., Zhao, X., Saini, H. S., ... & Dennis, E. S. (2005). Cold‐induced repression of the rice anther‐specific cell wall invertase gene OSINV4 is correlated with sucrose accumulation and pollen sterility. *Plant, Cell & Environment*, *28*(12), 1534-1551.

Oliver, S. N., Dennis, E. S., & Dolferus, R. (2007). ABA regulates apoplastic sugar transport and is a potential signal for cold-induced pollen sterility in rice. *Plant and Cell physiology*, *48*(9), 1319-1330.

Qin, P., Tu, B., Wang, Y., Deng, L., Quilichini, T. D., Li, T., ... & Li, S. (2012). ABCG15 encodes an ABC transporter protein, and is essential for post-meiotic anther and pollen exine development in rice. *Plant and Cell Physiology*, *54*(1), 138-154.

Shi, J., Tan, H., Yu, X. H., Liu, Y., Liang, W., Ranathunge, K., ... & Shanklin, J. (2011). Defective pollen wall is required for anther and microspore development in rice and encodes a fatty acyl carrier protein reductase. *The Plant Cell*, *23*(6), 2225-2246.

Shi, X., Sun, X., Zhang, Z., Feng, D., Zhang, Q., Han, L., ... & Lu, T. (2014). GLUCAN SYNTHASE-LIKE 5 (GSL5) plays an essential role in male fertility by regulating callose metabolism during microsporogenesis in rice. *Plant and Cell Physiology*, *56*(3), 497-509.

Sumiyoshi, M., Inamura, T., Nakamura, A., Aohara, T., Ishii, T., Satoh, S., & Iwai, H. (2014). UDP-arabinopyranose mutase 3 is required for pollen wall morphogenesis in rice (Oryza sativa). *Plant and Cell Physiology*, *56*(2), 232-241.

Tan, H., Liang, W., Hu, J., & Zhang, D. (2012). MTR1 encodes a secretory fasciclin glycoprotein required for male reproductive development in rice. *Developmental cell*, *22*(6), 1127-1137.

Ueda, K., Yoshimura, F., Miyao, A., Hirochika, H., Nonomura, K. I., & Wabiko, H. (2013). COLLAPSED ABNORMAL POLLEN1 gene encoding the arabinokinase-like protein is involved in pollen development in rice. *Plant physiology*, *162*(2), 858-871.

Wan, L., Zha, W., Cheng, X., Liu, C., Lv, L., Liu, C., ... & He, G. (2011). A rice β-1, 3-glucanase gene Osg1 is required for callose degradation in pollen development. *Planta*, *233*(2), 309-323.

Wang, A., Xia, Q., Xie, W., Datla, R., & Selvaraj, G. (2003). The classical Ubisch bodies carry a sporophytically produced structural protein (RAFTIN) that is essential for pollen development. *Proceedings of the National Academy of Sciences*, *100*(24), 14487-14492.

Wang, Y., Lin, Y. C., So, J., Du, Y., & Lo, C. (2013). Conserved metabolic steps for sporopollenin precursor formation in tobacco and rice. *Physiologia plantarum*, *149*(1), 13-24.

Yang, X., Wu, D., Shi, J., He, Y., Pinot, F., Grausem, B., ... & Liang, W. (2014). Rice CYP703A3, a cytochrome P450 hydroxylase, is essential for development of anther cuticle and pollen exine. *Journal of integrative plant biology*, *56*(10), 979-994.

Yang, L., Qian, X., Chen, M., Fei, Q., Meyers, B. C., Liang, W., & Zhang, D. (2016). Regulatory role of OsTDL1A-MSP1 signaling in specifying anther cell identity in rice. *Plant Physiology, 171*(3), 2085-2100.

Yang, Z., Sun, L., Zhang, P., Zhang, Y., Yu, P., Liu, L., ... & Cao, L. (2019). TDR INTERACTING PROTEIN 3, encoding a PHD‐finger transcription factor, regulates Ubisch bodies and pollen wall formation in rice. *The Plant Journal, 99*(5)*,* 844-861.

Yi, J., Moon, S., Lee, Y. S., Zhu, L., Liang, W., Zhang, D., ... & An, G. (2016). Defective tapetum cell death 1 (DTC1) regulates ROS levels by binding to metallothionein during tapetum degeneration. *Plant physiology*, *170*(3), 1611-1623.

Yu, J., Han, J., Kim, Y. J., Song, M., Yang, Z., He, Y., ... & Zhang, D. (2017). Two rice receptor-like kinases maintain male fertility under changing temperatures. *Proceedings of the National Academy of Sciences*, *114*(46), 12327-12332.

Zhang, H., Liang, W., Yang, X., Luo, X., Jiang, N., Ma, H., & Zhang, D. (2010a). Carbon starved anther encodes a MYB domain protein that regulates sugar partitioning required for rice pollen development. *The Plant Cell*, *22*(3), 672-689.

Zhang, S., Fang, Z., Zhu, J., Gao, J., & Yang, Z. (2010b). OsMYB103 is required for rice anther development by regulating tapetum development and exine formation. *Chinese Science Bulletin*, *55*(29), 3288-3297.

Zhang, D., Liang, W., Yin, C., Zong, J., Gu, F., & Zhang, D. (2010c). OsC6, encoding a lipid transfer protein, is required for postmeiotic anther development in rice. *Plant physiology*, *154*(1), 149-162.

Zhao, G., Shi, J., Liang, W., Xue, F., Luo, Q., Zhu, L., ... & Zhang, D. B. (2015). Two ATP Binding Cassette G (ABCG) transporters, OsABCG26 and OsABCG15, collaboratively regulate rice male reproduction. *Plant Physiology 169*(3), 2064-2079.

Zhu, L., Shi, J., Zhao, G., Zhang, D., & Liang, W. (2013). Post-meiotic deficient anther1 (PDA1) encodes an ABC transporter required for the development of anther cuticle and pollen exine in rice. *Journal of Plant Biology*, *56*(1), 59-68.

Zhu, X., Yu, J., Shi, J., Tohge, T., Fernie, A. R., Meir, S., ... & Liang, W. (2017). The polyketide synthase OsPKS2 is essential for pollen exine and Ubisch body patterning in rice. *Journal of integrative plant biology*, *59*(9), 612-628.
